# Supplementary material for: Mucosal Vaccination with UV-Inactivated Chlamydia suis in Pre-Exposed Outbred Pigs Decreases Pathogen Load and Induces CD4 T-Cell Maturation into IFN-γ+ Effector Memory Cells
Source: Vaccines (Basel). 2020 Jul 2;8(3):353. doi: 10.3390/vaccines8030353 (PMC7564508; doi:10.3390/vaccines8030353)
Supplement: Supplementary file 1 [file vaccines-08-00353-s001.zip › Amaral et al, 2020_Sup Figure S2.pdf]

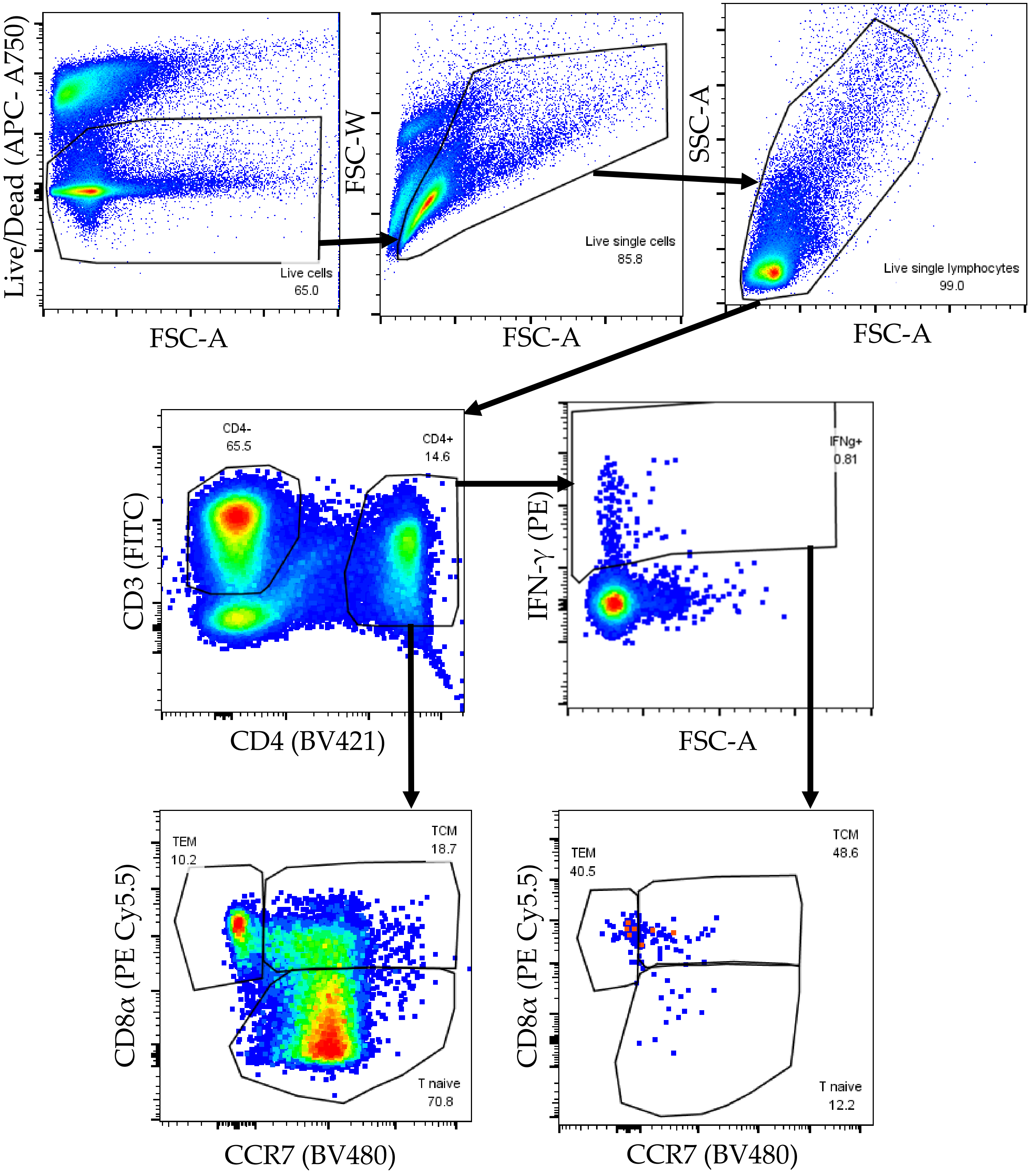

Supplementary Figure S2. Gating hierarchy for PBMC intracellular cytokine analysis via flow cytometry. Cytokine production in CD4$^+$ T-cells was analyzed via polychromatic flow cytometry. Live cells were identified by a gate on Live/Dead Near infra-red staining. Next, doublets were excluded by an FSC-A (area)/FSC-W (width) gate. Lymphocytes were then identified by cell size (FSC-A) and granularity (SSC-A). CD4$^+$ T-cells were identified via CD3 and CD4 markers and IFN-γ production was analyzed in live single CD4$^+$ T cells. CD4$^+$ and IFN-γ$^+$ CD4$^+$ cells were further differentiated into naïve, central and effector memory cells (T naïve, T_{CM}, T_{EM}, respectively) via CCR7 and CD8α markers. T_{CM} is CCR7$^+$CD8α$^+$ and T_{EM} is CCR7$^-$CD8α$^+$. Gating is based on the relevant fluorescent minus one (FMO) controls.
