# Supplementary material for: Mucosal Vaccination with UV-Inactivated Chlamydia suis in Pre-Exposed Outbred Pigs Decreases Pathogen Load and Induces CD4 T-Cell Maturation into IFN-γ+ Effector Memory Cells
Source: Vaccines (Basel). 2020 Jul 2;8(3):353. doi: 10.3390/vaccines8030353 (PMC7564508; doi:10.3390/vaccines8030353)
Supplement: Supplementary file 1 [file vaccines-08-00353-s001.zip › Amaral et al, 2020_Sup Figure S1.pdf]

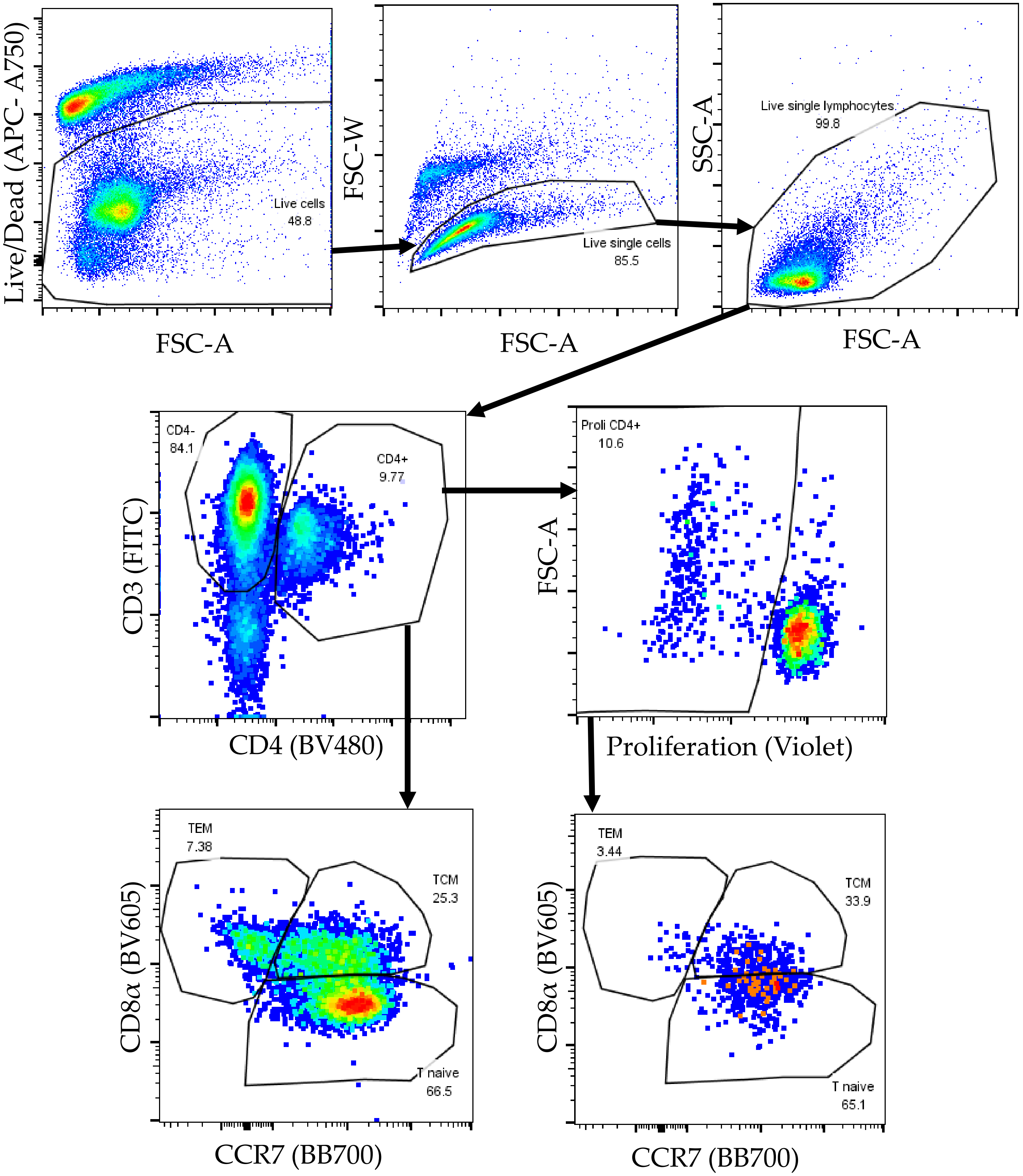

Supplementary Figure S1. Gating hierarchy for PBMC proliferation analysis via flow cytometry. Proliferation of CD4$^+$ T cells was analyzed by CellTrace^{TM} Violet in combination with FCM stainings for immune markers. Live cells were identified by a gate on Live/Dead Near infra red staining. Next, doublets were excluded by an FSC-A (area)/FSC-W (width) gate. Lymphocytes were then identified by cell size (FSC-A) and granularity (SSC-A). CD4$^+$ T cells were identified via their CD3 and CD4 expression. Within these CD4$^+$ T cells, proliferation was analyzed using a CellTrace^{TM} Violet/FSC-A gate. CD4$^+$ and proliferating CD4$^+$ cells were further differentiated into naïve (T naïve), central (T_{CM}) and effector memory cells (T_{EM}) via CCR7 and CD8α markers: T_{CM} is CCR7$^+$CD8α^+ and T_{EM} is CCR7$^-$CD8α^+. Gating is based on relevant fluorescent minus one (FMO) controls.
