# Supplementary material for: Mucosal Vaccination with UV-Inactivated Chlamydia suis in Pre-Exposed Outbred Pigs Decreases Pathogen Load and Induces CD4 T-Cell Maturation into IFN-γ+ Effector Memory Cells
Source: Vaccines (Basel). 2020 Jul 2;8(3):353. doi: 10.3390/vaccines8030353 (PMC7564508; doi:10.3390/vaccines8030353)
Supplement: Supplementary file 1 [file vaccines-08-00353-s001.zip › Amaral et al, 2020_Sup Figure S4.pdf]

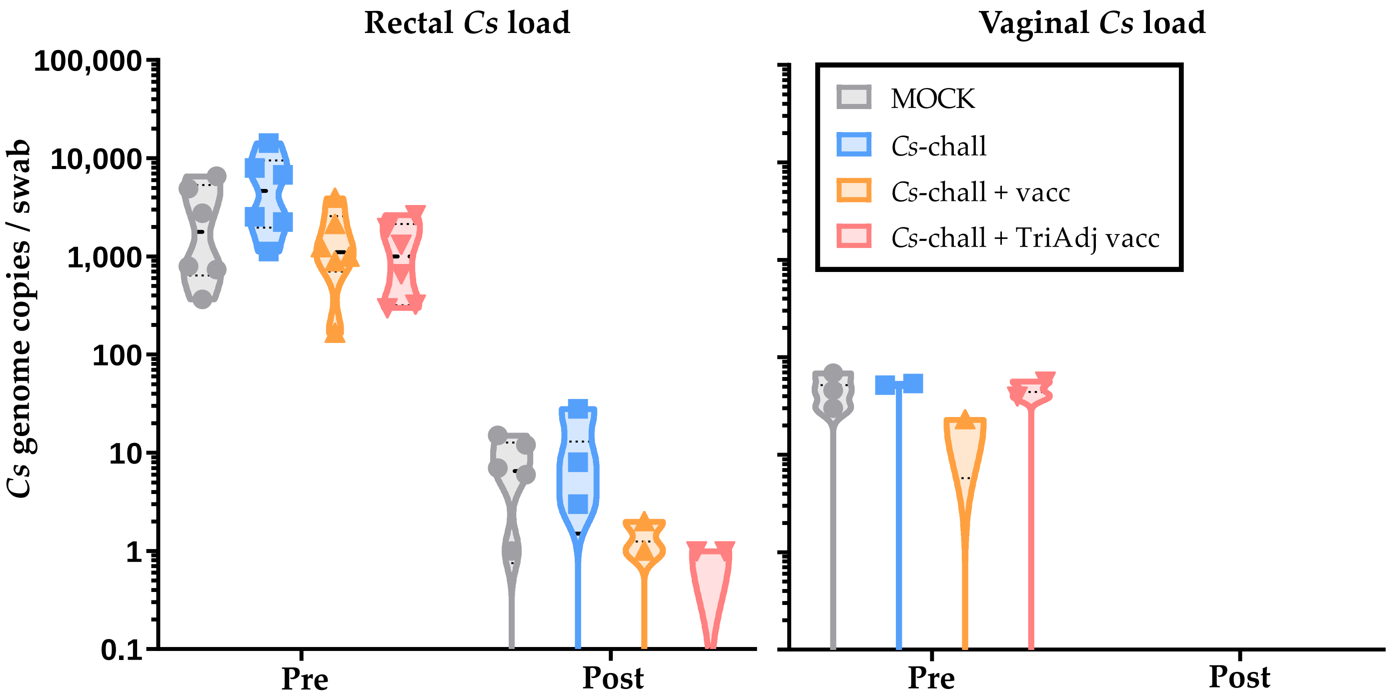

Supplementary Figure S4. Antibiotic treatment eliminates vaginal *C. suis* load and decreases rectal *C. suis* load. The *C. suis* load in rectal and vaginal swabs from MOCK (gray), *C. suis* challenged (*Cs*-chall, blue), *C. suis* challenged + vaccinated (*Cs*-chall + vacc, orange) and *C. suis* challenged + Tri Adjuvant vaccinated (*Cs*-chall + TriAdj vacc, red) pigs were analyzed via qPCR prior to challenge (Pre, x-axis) and post challenge (Post, x-axis). Chlamydia levels below the standard curve were considered negative and are not represented in the graph. Values for individual pigs, medians, and 25/75 percentiles are shown.
