# Supplementary material for: Mucosal Vaccination with UV-Inactivated Chlamydia suis in Pre-Exposed Outbred Pigs Decreases Pathogen Load and Induces CD4 T-Cell Maturation into IFN-γ+ Effector Memory Cells
Source: Vaccines (Basel). 2020 Jul 2;8(3):353. doi: 10.3390/vaccines8030353 (PMC7564508; doi:10.3390/vaccines8030353)
Supplement: Supplementary file 1 [file vaccines-08-00353-s001.zip › Amaral et al, 2020_Sup Figure S3.pdf]

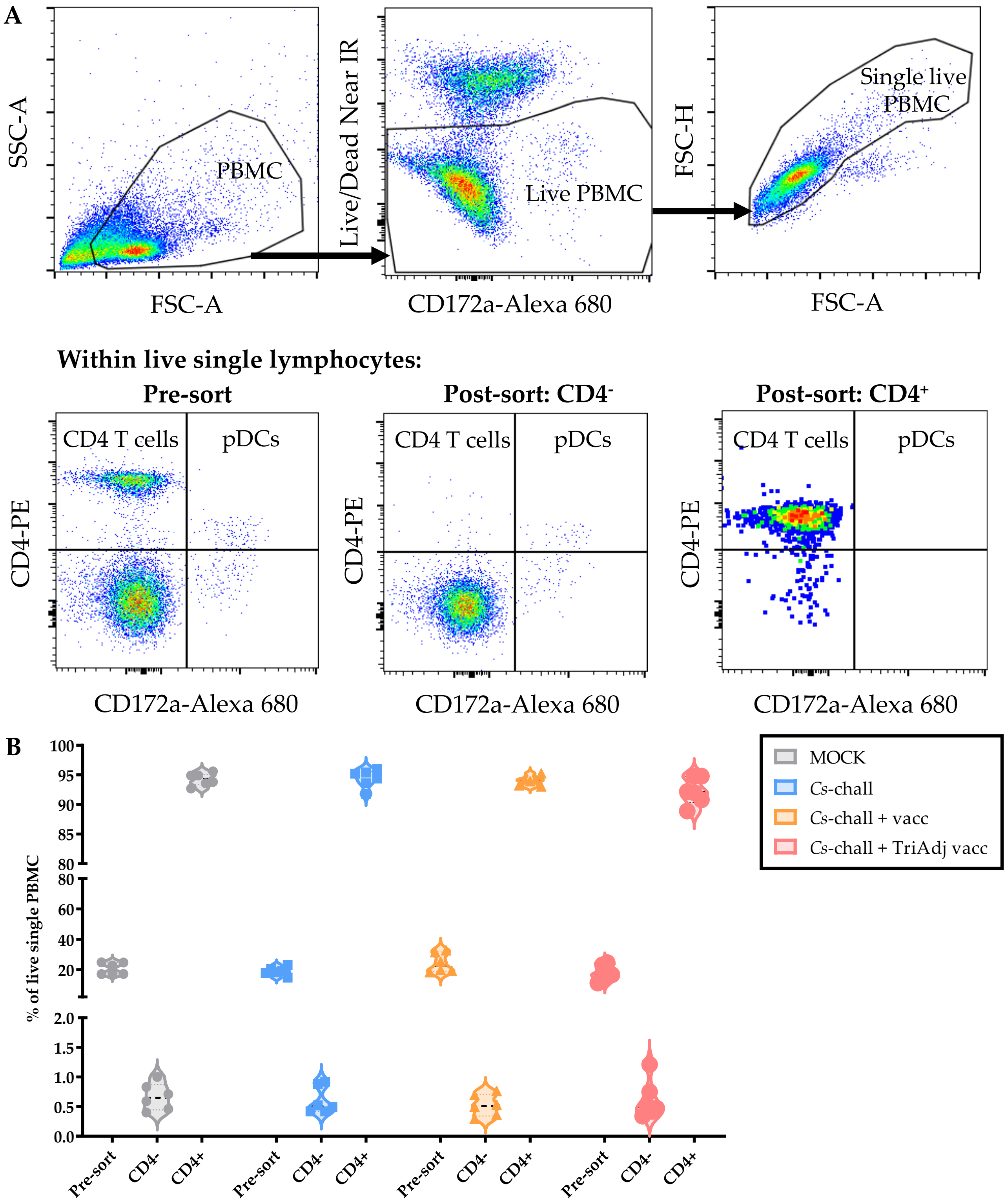

Supplementary Figure S3. Reanalysis of blood CD4 T cells isolated by magnetic activated cell sorting via flow cytometry. PBMC from 37 days post first vaccination were isolated from MOCK (gray), *C. suis* challenged (*Cs*-chall, blue), *C. suis* challenged + vaccinated (*Cs*-chall + vacc, orange) and *C. suis* challenged + Tri Adjuvant vaccinated (*Cs*-chall + TriAdj vacc, red) pigs. PBMC were cultured with *C. suis* lysate overnight and CD4 T cells were isolated by magnetic activated cell sorting (MACS) using positive cell sorting on $CD4^+$ cells (Miltenyi Biotech), according to manufacturer's protocol. MACS sorts were then identified via flow cytometry. (A) Gating hierarchy of $CD4^+$ cells: Lymphocytes were identified by cell size (FSC-A) and granularity (SSC-A); live cells were then identified by Live/Dead Near infra-red and CD172a staining; next, doublets were excluded by an FSC-A (area)/FSC-H (height) gate; CD4 T-cells were identified as $CD4^+CD172a^-$ cells, as oppose to plasmacytoid dendritic cells (pDC) which are $CD4^+CD172a^+$ cells. Gating is based on the relevant fluorescent minus one (FMO) controls. (B) The purity of the MACS sorts was confirmed by flow cytometry reanalysis of the sorted fractions. Values for individual pigs, medians, and 25/75 percentiles are shown.
