# Supplementary material for: Mucosal Vaccination with UV-Inactivated Chlamydia suis in Pre-Exposed Outbred Pigs Decreases Pathogen Load and Induces CD4 T-Cell Maturation into IFN-γ+ Effector Memory Cells
Source: Vaccines (Basel). 2020 Jul 2;8(3):353. doi: 10.3390/vaccines8030353 (PMC7564508; doi:10.3390/vaccines8030353)
Supplement: Supplementary file 1 [file vaccines-08-00353-s001.zip › Amaral et al, 2020_Sup Figure S5.pdf]

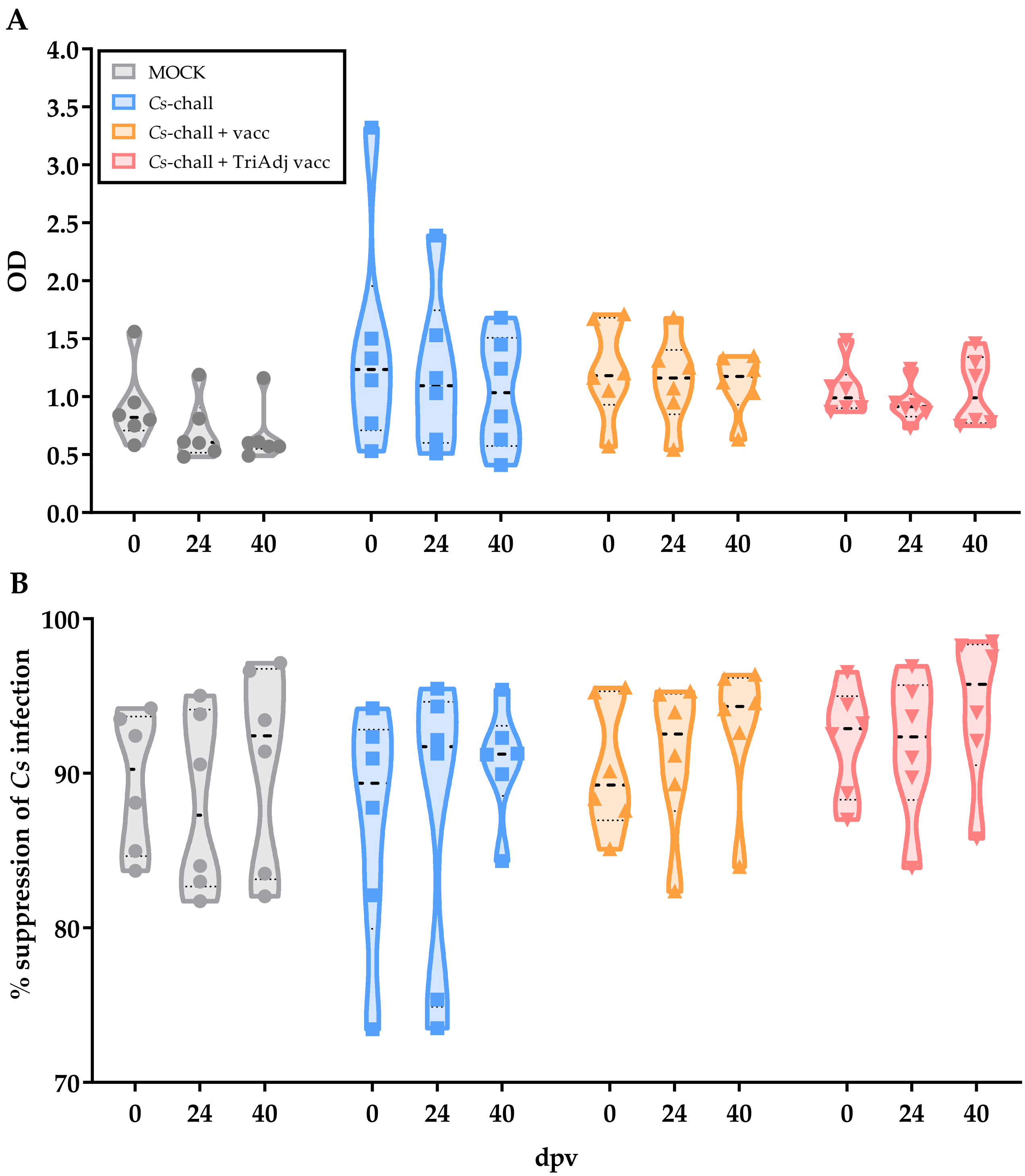

Supplementary Figure S5. Serum anti-*C. suis* antibody levels in commercial pigs from high-health farms. The humoral immune response was analyzed in MOCK (gray), *C. suis* challenged (*Cs*-chall, blue), *C. suis* challenged + vaccinated (*Cs*-chall + vacc, orange) and *C. suis* challenged + Tri Adjuvant vaccinated (*Cs*-chall + TriAdj vacc, red) pigs. (A) Anti-*C. suis* serum IgG levels at stated days post first vaccination (dpv, x-axis) were analyzed via peptide ELISA. (B) Neutralizing antibodies in serum were analyzed by infecting HeLa cells with *Cs* in the presence of serum (1:10 dilution). The % suppression of infection (y axis) was calculated via flow cytometry by dividing the infection rate at the day post first vaccination (dpv, x-axis) by the infection rate of serum from specific pathogen free (SPF) pigs. Values for individual pigs, medians, and 25/75 percentiles are shown.
