# Supplementary material for: Mucosal Vaccination with UV-Inactivated Chlamydia suis in Pre-Exposed Outbred Pigs Decreases Pathogen Load and Induces CD4 T-Cell Maturation into IFN-γ+ Effector Memory Cells
Source: Vaccines (Basel). 2020 Jul 2;8(3):353. doi: 10.3390/vaccines8030353 (PMC7564508; doi:10.3390/vaccines8030353)
Supplement: Supplementary file 1 [file vaccines-08-00353-s001.zip › Amaral et al, 2020_Sup Table S1.pdf]

Supplementary Table S1. Blood CD4$^+$ T cell total mRNAs profiled using RNA-seq. The pairwise differential gene expression analysis between non-challenged MOCK and challenged groups (*C. suis* challenged, *C. suis* challenged + vaccinated and *C. suis* challenged + Tri Adjuvant vaccinated) was assessed using DESeq2. Significance of the canonical pathway was assessed using Fisher's exact test. All multiple testing was adjusted by Benjamini-Hochberg.

Ensemble Gene ID	Gene Symbol	Gene Name	General Function	Log2 Fold Change	Adjusted p value
ENSSSCG00000026842	CDH18	cadherin 18	adhesion	6.514001499	0.003218955
ENSSSCG00000000857	IGF1	insulin-like growth factor 1	growth and proliferation	6.495027138	0.002942227
ENSSSCG00000009502	UGGT2	UDP-glucose glycoprotein glucosyltransferase 2	metabolism	6.09677358	0.002983538
ENSSSCG00000027678	CCDC175	coiled-coil Domain Containing 175	structure and trafficking	5.919923343	0.008648947
ENSSSCG00000032365	GPR87	G-protein coupled receptor 87	communication and migration	5.828547485	0.019395021
ENSSSCG00000012002	ROBO2	roundabout guidance receptor 2	proliferation and migration	5.711321741	0.005599321
ENSSSCG00000035000	ABCA16	ATP-binding cassete sub-family A, member 16	molecular transport	5.540309545	0.01728579
ENSSSCG00000016554	MEST	mesoderm specific transcript	metabolism	5.320303203	0.02209914
ENSSSCG00000009217	PKD2	polycystin 2	ion channel	5.213497139	0.024469655
ENSSSCG00000013382	PLEKHA7	pleckstrin homology domain containing A7	adhesion	5.146365546	0.015107422
ENSSSCG00000030877	FRMPD4	FERM and PDZ domain containing 4	scaffolding	5.140995088	0.003574795
ENSSSCG00000017261	ARSG	arylsulfatase G	metabolism	5.121888326	0.015931799
ENSSSCG00000022741	PDGFRB	platelet dervied growth factor receptor beta	growth	5.110208465	0.037526456
ENSSSCG00000034000	SERPINB11L1 [1]	Serpin-like B11	metabolism	5.008991958	0.044098118
ENSSSCG00000007675	EPHB4	ephrin type-B receptor	proliferation	4.81030992	0.041831729
ENSSSCG00000002277	SPTB	spectrin beta	membrane stability	4.795997278	0.018496517
ENSSSCG00000005762	KCNT1	potassium channel subfamily T, member 1	ion channel	4.690706608	0.028148799
ENSSSCG00000003251	NLRP12	NLR family pyrin domain containing 12	inflammation	4.57693532	0.038584841
ENSSSCG00000011485	PTPRG	protein tyrosine phosphatase receptor type G	growth and migration	4.514900161	0.029148929
ENSSSCG00000003789	CTH	cystathionine gamma-lyase	metabolism	4.484348504	0.006405136
ENSSSCG00000016678	NOD1	nucleotide binding oligomerization domain containing 1	inflammation	4.388027764	0.0035279
ENSSSCG00000010626	RBM20	RNA binding motif 20	alternative splicing	4.382216306	0.024142368
ENSSSCG00000039854	TLCD4	TLC domain containing 4	metabolism	4.339526312	0.04830757
ENSSSCG00000036553	SAMD12	sterile alpha motif domain containing 12	DNA binding	4.184921478	0.025461995
ENSSSCG00000032946	HMGN5	high mobility group nueclosome binding domain 5	DNA binding	4.144044731	0.029480236
ENSSSCG00000040967	NACC2	nuclus accumbens-associated protein 2	transcriptional regulation	4.068007871	0.046812872
ENSSSCG00000021562	PLXNA4	plexin A4	cytoskeleton remodeling	4.041299219	0.018980378
ENSSSCG00000009429	TNFSF11	tumor necrosis factor ligand superfamily member 11	inflammation and activation	4.011210923	0.020315766
ENSSSCG00000013147	FAM111B	family with sequence similiarity 111 member B	metabolism	3.941219485	0.016870537
ENSSSCG00000008761	TBC1D19	TBC1 domain family member 19	signaling and metabolism	3.932766694	0.011187952
ENSSSCG00000014625	TRIM3	tripartite motif containing 3	molecular transport	3.87494137	0.025398417
ENSSSCG00000020864	VDR	vitamin D3 receptor	metabolism and inflammation	3.816828275	0.036776221
ENSSSCG00000011353	PFKFB4	6-phosophofructo-2-kinase/fructose-2,6-biphosphatase 4	metabolism	3.81025969	0.018098585
ENSSSCG00000014179	FAM174A	family with sequence similiarity 174 member A	signaling	3.757174913	0.038221344
ENSSSCG00000024065	TMTC2	transmembrane O-mannosyltransferase targeting conditions 2	metabolism	3.749174547	0.040946516
ENSSSCG00000032086	ZNF583	zinc finger protein 583	transcriptional regulation	3.721583593	0.007057135
ENSSSCG00000035258	RABL3	member of RAS oncogene family like 3	signaling	3.700079683	0.002271332
ENSSSCG00000037475	CTSG	cathepsin G	inflammation	3.581407665	0.024589968
ENSSSCG00000015808	ADAM9	disintegrin and metalloproteinase domain-containg protien 9	adhesion	3.426658472	0.018098585
ENSSSCG00000011755	NCEH1	neutral cholesterol ester hydrolase 1	metabolism	3.407326519	0.008197997
ENSSSCG00000037420	TMEM170B	transmembrane protein 170B	signaling	3.37910639	0.049163621
ENSSSCG00000022131	KCTD6	potassium channel tetramerization domain containg 6	signaling	3.359860673	0.020781425
ENSSSCG00000031847	RBM28	RNA binding motif 28	transcriptional regulation	3.336667366	0.012149562
ENSSSCG00000012172	ACOT9	acyl-CoA thioesterase 9	metabolism	3.315946983	0.032564652

Supplementary Table S1 (CONTINUATION). Blood CD4⁺ T cell total mRNAs profiled using RNA-seq. The pairwise differential gene expression analysis between non-challenged MOCK and challenged groups (*C. suis* challenged, *C. suis* challenged + vaccinated and *C. suis* challenged + Tri Adjuvant vaccinated) was assessed using DESeq2. Significance of the canonical pathway was assessed using Fisher's exact test. All multiple testing was adjusted by Benjamini-Hochberg.

Ensemble Gene ID	Gene Symbol	Gene Name	General Function	Log2 Fold Change	Adjusted p value
ENSSSCG00000013861	SLC35E1	solute carrier family 35 member E1	molecular transport	3.314660992	0.00877453
ENSSSCG00000038493	PFDN1	prefolding subunit 1	molecular transport	3.263456994	0.026294999
ENSSSCG00000035193	MRPS23	mitochondrial ribosomal protein S23	survival	3.162095174	0.048396984
ENSSSCG00000037325	UBIAD1	ubiA prenyltransferase domain containing 1	metabolism	3.127636805	0.027705123
ENSSSCG00000005011	NEMF	nuclear export mediator factor	molecular transport	3.113457185	0.004908871
ENSSSCG00000002305	EXD2	exonuclease 3'-5' domain containing 2	DNA repair	3.088641479	0.032943414
ENSSSCG00000015892	PSMD14	proteasome 26S subunit, non-ATPase 14	protein degradation	3.054264429	0.016268598
ENSSSCG00000033162	UBAC1	ubiquitin-associated domain-containing 1	protein degradation	3.025612433	0.0122442
ENSSSCG00000009193	SMARCAD1	SWI/SNF-related, matrix-associated actin-dependent regulator of chromatin, subfamily A, containing DEAD/H Box 1	DNA binding	3.001023959	0.002894647
ENSSSCG00000016226	FARSB	phenylalanyl-tRNA synthetase beta chain	metabolism	2.994310907	0.00371746
ENSSSCG00000012131	MOSPD2	motile sperm domain containing 2	metabolism	2.9913784	0.006024667
ENSSSCG00000009305	GTF3A	general transcription factor 3A	transcription	2.987510154	0.036921829
ENSSSCG00000028345	ZNF33B	zing finger protein 33B	signaling and metabolism	2.966858443	0.015945452
ENSSSCG00000004946	ZWILCH	zwilch kinetochore protein	proliferation	2.92507877	0.036921829
ENSSSCG00000032580	MGST1	microsomal glutathione s-transferase 1	metabolism	2.89902142	0.039963149
ENSSSCG00000000844	NFYB	nuclear transcription factor Y subunit beta	transcriptional regulation	2.893050892	0.019602372
ENSSSCG00000002525	TRAF3	TNF receptor associated factor 3	signaling and inflammation	2.885270408	0.029148929
ENSSSCG00000032919	ATG4B	autophagy related 4B cysteine peptidase	autophagy	2.821028399	0.032943414
ENSSSCG00000002937	ZNF420	zing finger protein 420	signaling and metabolism	2.793284015	0.039273246
ENSSSCG00000020885	NUS1	nuclear undecaprenyl pyrophosphate synthetae 1	metabolism	2.769905114	0.02991454
ENSSSCG00000005507	PSMD5	proteasome 26S subunit, non-ATPase 5	protein degradation	2.758698463	0.048977939
ENSSSCG00000005587	NEK6	NIMA related kinase 6	signaling and proliferation	2.717168935	0.04243732
ENSSSCG00000000964	LMF2	lipase maturation factor 2	metabolism	2.685857445	0.031616857
ENSSSCG00000003506	PINK1	PTEN induced kinase 1	signaling	2.662957517	0.045979115
ENSSSCG00000010949	DAPK1	death associated protein kinase 1	signaling and surival	2.635847767	0.029957851
ENSSSCG00000012424	ABCB7	ATP-binding cassete sub-family B, member 7	molecular transport	2.494990364	0.015843827
ENSSSCG00000033295	EIF2S1	eukaryotic translation initiation factor 2 subunit alpha	protein synthesis	2.474632908	0.009531644
ENSSSCG00000011162	LARP4B	La ribonucleoprotein 4B	DNA binding	2.454986159	0.044627204
ENSSSCG00000001667	ZNF318	zing finger protein 318	signaling and metabolism	2.449922752	0.046620743
ENSSSCG00000015590	FLVCR1	feline leukemia virus subgroup C receptor-related 1	molecular transport	2.396401517	0.038596864
ENSSSCG00000013347	PRMT3	protein arginine methyltransferase 3	metabolism	2.357566489	0.033986845
ENSSSCG00000036790	AKAP7	A-kinase anchoring protein	signaling	2.356699873	0.037294235
ENSSSCG00000038895	DCTN6	dynactin subunit 6	molecular transport	2.345543214	0.031545264
ENSSSCG00000033154	ARL6IP5	ADP ribosylation factor like GTPase 6 interacting protein 5	signaling	2.341746948	0.012797427
ENSSSCG00000006346	ATF6	activating transcription factor 6	transcription	2.309316884	0.033964553
ENSSSCG00000002844	PHKB	phosphorylase kinase regulatory subunit beta	signaling	2.294356394	0.01654099
ENSSSCG00000010874	ADSS	adenylsuccinate synthase 2	metabolism	2.272571211	0.031666077
ENSSSCG00000004576	RORA	RAR related orphan recetor A	transcriptional regulation	2.240683079	0.017383661
ENSSSCG00000040954	CSTF3	cleavage stimulation factor subunit 3	transcriptional regulation	2.19786242	0.039135766
ENSSSCG00000004999	MIS18BP1	MIS18 binding protein	DNA binding	2.178375045	0.03583276
ENSSSCG00000011833	DLG1	discs large MAGUK scaffold protein 1	signaling	2.003275128	0.027896597
ENSSSCG00000032357	APPL1	adaptor protein, phosphotyrosine interacting with PH domain and leucine zipper 1	proliferation	1.92155645	0.037191961
ENSSSCG00000002701	MON1B	MON1 homolog B	molecular transport	1.772512299	0.048396984
ENSSSCG00000033115	IFNAR1	interferon alpha and beta subunit 1	signaling and inflammation	1.572971046	0.017506452
ENSSSCG00000006553	UBAP2L	Ubiquitin-associated protein 2 L	protein degradation	1.177037687	0.039579458

[1] no human ortholog
